# Digestive Ferments

**Published:** 1885-09

**Authors:** 


DIGESTIVE FERMENTS.

The following extract is also from the *British Medical Journal:*

"The association was fortunate in securing the services of so eminently practical a pharmacologist as Dr. William Roberts, of Manchester, to deliver the address in therapeutics at Cardiff this year. Dr. Roberts has succeeded in establishing for himself a world-wide reputation, not only as a physiologist, but as a clinical teacher, and his work always commands attention and interest. His Lumleian lectures on the digestive ferments, delivered before the Royal College of Physicians of London in

1880, are usually regarded as a type of good scientific work. His address before the association, on the feeding of the sick, although more restricted in its scope, has been even more widely read, and has excited much comment, both in the medical and in the general press. We are glad to find that Dr. Roberts intends publishing, shortly, the results of the experiments on which he has been long engaged respecting the influence of salivary and peptic digestion on the "accessories," alcoholic beverages, tea, coffee, cocoa, etc., which are universally employed in one form or another as food. This is an inquiry of an eminently practical nature, the importance of which it would be difficult to overestimate. It is clearly a subject closely affecting the welfare of our patients, and one which must of necessity daily occupy the attention of every physician. Observations of this class, fortunately, do not necessitate the employment of expensive apparatus, and can be carried out almost as well in the consulting room as in the physiological laboratory. We believe that every medical man would do well to make a point of testing for himself the activity of the digestive ferments which he is in the habit of prescribing. It is a subject which will probably occupy the attention of the Collective Investigation Committee at no distant date. Dr. Roberts speaks of having recently had placed at his disposal a preparation, free from taste and smell, consisting of the pancreatic enzymes in a highly purified state. It is not hygroscopic, and may be kept unchanged for an indefinite period, freely exposed to the air. A substance of this nature, in the form of a white powder, was prepared some years ago by Fairchild, the American chemist, and has already been extensively used in this country. The pancreatic juice as is now well known, consists of four ferments: trypsin, which changes proteids into peptones in alkaline and neutral media; a curdling ferment which curdles the casein of milk; the pancreatic diastase, which acts like extract of malt, changing starch into sugar and dextrine; and an emulsive ferment which emulsifies and partially saponifies fats. There can be no doubt that, as a digestive agent, extract of pancreas is vastly superior to any preparation made from gastric juice. "The pancreas," says Dr. Roberts, "excels the stomach as a digestive organ, in that it has has power to digest the two great alimentary principles, starch and proteids, and an extract of the gland is possessed of similar endowments." There can be no doubt that a series of carefully reported cases of different diseases treated by the pancreatic method of predigestion is a desideratum. It has proved useful in many hands in uræmic vomiting, gastric catarrh, pernicious anæmia, gastric ulcer, and pyloric and intestinal obstruction. Its introduction has probably done more than any other therapeutic measure of recent times to lessen infant mortality.

